# 1042. Safety of Investigational Microbiota-Based Live Biotherapeutic RBX2660 in Individuals with Recurrent *Clostridioides difficile* Infection: Data from Five Prospective Clinical Studies

**DOI:** 10.1093/ofid/ofab466.1236

**Published:** 2021-12-04

**Authors:** Tricia Braun, Beth Guthmueller, Adam J Harvey

**Affiliations:** 1 Rebiotix, A Ferring Company, Roseville, Minnesota; 2 Rebiotix Inc, A Ferring Company, Victoria, Minnesota

## Abstract

**Background:**

Microbiota-based treatments have shown promise to reduce recurrence, morbidity, and mortality for recurrent *Clostridioides difficile* infections (rCDI), but consistent and reliable safety data are needed to support regulatory approvals and broaden patient access. Here we provide cumulative safety data from 5 prospective clinical studies evaluating RBX2660—a standardized, microbiota-based investigational live biotherapeutic—for reducing rCDI.

**Methods:**

This analysis included three Phase 2 (PUNCH CD, PUNCH CD2, PUNCH CD Open Label) and two Phase 3 trials (PUNCH CD3, PUNCH CD3-OLS *ad hoc* analysis). Participants were ≥18 years old with documented rCDI who completed standard-of-care oral antibiotic therapy prior to treatment with RBX2660. PUNCH CD3-OLS allowed participants with comorbidities of irritable bowel syndrome (IBS) or inflammatory bowel disease (IBD). Depending on the trial, assigned study treatment was 1 or 2 doses of RBX2660 (or placebo), administered rectally. Participants whose CDI recurred within 8 weeks were eligible for additional RBX2660 treatment. Treatment-emergent adverse events (TEAEs) were recorded for at least 6 months following last study treatment; CD2 and CD Open Label recorded TEAEs for 24 months.

**Results:**

Among 620 participants who received at least one RBX2660 dose (assigned treatment or after recurrence), 324 (52.3%) received 1, 270 (43.5%) received 2, 14 (2.3%) received 3, and 12 (1.9%) received 4. 83 participants received blinded placebo only. A total of 1980 TEAEs were reported from 432 (69.7%) RBX2660-treated participants, compared to 174 TEAEs in 50 (60.2%) placebo-only treated participants. Most TEAEs were mild or moderate in severity, with diarrhea common in all treatment groups. No potentially life-threatening TEAEs were considered related to RBX2660. Study discontinuation due to TEAEs was minimal (< 1%) with none related to RBX2660. There were no reported infections for which the causative pathogen was traced to RBX2660.

**Conclusion:**

Across five clinical studies with consistent investigational product, RBX2660 was well-tolerated in rCDI participants. In aggregate, this data provides compelling and consistent safety data for RBX2660.

**Disclosures:**

**Tricia Braun, PharmD**, **Rebiotix, a Ferring Company** (Employee) **Beth Guthmueller, AS**, **Rebiotix Inc, A Ferring Company** (Employee) **Adam J. Harvey, PhD**, **Rebiotix, A Ferring Company** (Employee)

